# Senators in Motley

**Published:** 1891-03

**Authors:** 


### The Medical Bill Ridiculed in the Senate.

Since the article "Hope Deferred" was put in type, strange things have happened at the capitol.

The medical bill was called up by Senator Pope on the 20th inst. No sooner was it read than certain Senators proceeded to cover themselves all over with glory (?) Senator B. moved an amendment, which, being adopted, gave the homeopaths and eclectics each three places on the board of examiners—to thiee physicians. This killed the bill. The committee of medical gentlemen, some of whom were present, in disgust, asked Senator Pope to move the indefinite postponement of the bill, as they could not submit to such injustice, and it was done. This effort in behalf of a long suffering public has thus been sent to join the long procession of similar efforts defeated by homeopathic assurance and misrepresentation. *Vive la Bagatelle!* Quackery has received another impetus and charlatanry reigns supreme throughout the land. These gentlemen thus go on record as endorsers, if not champions of the homeopathic cause and as champions of unequal rights. Disregarding the fact that this element constitutes but two per cent. of the practitioners of Texas, these gentlemen would give them 33⅓ per cent. representation in medical matters, matters in which we cannot see that they are entitled to any voice at all.

There appears to have been several Brutuses in this stabbing business. But the rididule that was hurled at the bill was the unkindest cut of all. Surely, any request emanating from so respectable a source, should have commanded respectful consideration.

The medical profession have heretofore consoled themselves with the reflection that the low average intelligence of previous legislatures had failed to grasp the broad humanitarian principles which underlie such efforts; and they had congratulated themselees that the twenty-second legislature was composed of a better order of men. Hence, the failure of the bill falls with a double force on them. That intelligent, well educated Senators, for the most part good lawyers, should fail to see the necessity for restrictions on a privilege, the indiscriminate exercise of which endangers human lives, is truly surprising. It would seem to be a self-evident proposition. That they should be hoodwinked by a handful of men who do not deny that while they call themselves homeopaths, they practice what they call "allopathy," the principles of which are directly antagonistic to homeopathy, and made to espouse a cause so empty and pretentious, is still more surprising; but that they should so far forge their own dignity, and the respect that is due the gentlemen represensing the State Medical Association, so far forget the essentials of good breeding as to stoop to ridicule in order to thwart a righteous and much needed reform, asked by those whom all recognize and respect as gentlemen, and the representatives of a large element of educated citizens, to say nothing of the people's petition, bearing fifteen thousand names—is indeed astonishing.

We learn with pain and mortification, that a certain popular Senator—the son-in-law of one of the most eminent and respected members of the medical profession—especially distinguished himself for facetiousness, and that a certain other gentleman, with a coarser article of ridicule, came in a good second in the "funny business."

We were not present; had we been, we should have blushed to see the honorable gentlemen so bemean themselves. We are told that it excelled the exploids of the famous "Bell of Cook" in the memorable Twenty-first. Senator Pope rebuked his hilarious colleagues and delared he was *ashamed of the action of the Senate!* All honor to him.

How are the mighty fallen! Once—to be a physician, if not to be "greater than a king"—was at least to occupy a position which commanded respect. To be a physician now, and to labor earnestly and conscientiously in the cause of humanity, to advocate a reform, by which life may be saved and suffering and wrong prevented, is to bring down the shafts of ridicule at the hands of brethren of the kindred profession of law; and even the gray hairs of veterans in the cause of humanity, will not protect from the witticisms (?) of conceited new-made Senators. Oh shame, where is thy blush!

In lieu of this bill, it is highly probable that the homeopathic bill, introduced by Mr. McKinney, will become the law It provides for a board of six medical censors, composed of the president and secretary of eaeh of the three "schools," and their sole functions is to pass upon the respectability of diplomas! What a farce. Were it not so late our committee might perhaps get the law of 1876,—the only one now on the statute of Texas,—patched up so as to make it operative, and it would do very well in absence of a better. And that step, indeed, is our only hope, even in the future. Some legislators seem to be unalterably opposed to any effort on the part of the physicians to "regulate,"—and are especially opposed to a central board.

[Since the above was in type the motion to postpone has been reconsidered; and Monday, March 23d, the bill is again up in the Senate.—Ed.]

